# Role of MicroRNAs in Parkinson’s Disease

**DOI:** 10.3390/ijms20225649

**Published:** 2019-11-12

**Authors:** Suh Yee Goh, Yin Xia Chao, Shaikali Thameem Dheen, Eng-King Tan, Samuel Sam-Wah Tay

**Affiliations:** 1 Department of Anatomy, Yong Loo Lin School of Medicine, National University of Singapore, 4 Medical Drive, Singapore 117594, Singapore; e0054068@u.nus.edu (S.Y.G.); anthead@nus.edu.sg (S.T.D.); 2 National Neuroscience Institute, 11 Jalan Tan Tock Seng, Singapore 308433, Singapore; 3 Department of Neurology, Singapore General Hospital, Outram Road, Singapore 169608, Singapore; 4 Medical Education, Research and Evaluation (MERE) department, Duke-NUS Medical School, 8 College Rd, Singapore 169857, Singapore; 5 Neuroscience and Behavioral Disorders (NBD) department, Duke-NUS Medical School, 8 College Rd, Singapore 169857, Singapore

**Keywords:** Parkinson’s disease, microRNAs, review

## Abstract

Parkinson’s disease (PD) is a disabling neurodegenerative disease that manifests with resting tremor, bradykinesia, rigidity and postural instability. Since the discovery of microRNAs (miRNAs) in 1993, miRNAs have been shown to be important biological molecules involved in diverse processes to maintain normal cellular functions. Over the past decade, many studies have reported dysregulation of miRNA expressions in PD. Here, we identified 15 miRNAs from 34 reported screening studies that demonstrated dysregulation in the brain and/or neuronal models, cerebrospinal fluid (CSF) and blood. Specific miRNAs-of-interest that have been implicated in PD pathogenesis include miR-30, miR-29, let-7, miR-485 and miR-26. However, there are several challenges and limitations in drawing definitive conclusions due to the small sample size in clinical studies, varied laboratory techniques and methodologies and their incomplete penetrance of the blood–brain barrier. Developing an optimal delivery system and unravelling druggable targets of miRNAs in both experimental and human models and clinical validation of the results may pave way for novel therapeutics in PD.

## 1. Introduction

Parkinson’s disease (PD) is the most common movement disorder in the aging population. An estimated prevalence of 1% of people above 60 years old suffer from PD [1]. PD involves a progressive loss of neurons in the brain, especially the loss of dopaminergic (DA) neurons in the substantia nigra pars compacta (SNc). When more than 50% to 70% of DA neurons are lost, resulting in the corresponding decrease in dopamine neurotransmitter production and signaling, this can manifest as motor dysfunctions and clinical symptoms such as resting tremor, bradykinesia, rigidity and postural instability [2]. In addition, intracellular inclusions, known as Lewy bodies, which are enriched with the aggregated α-synuclein protein (α-syn), are also often detected in the neurons of PD patients and are suggested to impair pathways such as vesicle trafficking or activating neuroinflammation [3]. In the Braak staging hypothesis, it was proposed that PD starts as a synucleinopathy in lower non-dopminergic structures of the brainstem and spreads rostrally to affect the SNc in the later stage of PD [4]. Aside from the SNc, PD-specific cell loss has also been reported in the ventral tegmental area, amygdala, and dorsal motor nucleus of the vagus nerve, hypothalamus, cortex, thalamus and many other brain regions [5]. It has been suggested that the loss of neurons in these areas may also contribute to the motor dysfunctions observed in PD as well as other non-related motor symptoms such as depression, anxiety and sleep disorders [6]. Therefore, in this review, we have considered studies that investigated not only the SNc, but also other brain regions. 

Currently, clinical treatments available for PD are mainly symptomatic which include medications such as Levodopa, dopamine agonists, catechol-O-methyl transferase (COMT) inhibitors and monoamine oxidase B inhibitors and non-pharmacological interventions such as deep brain stimulation [7,8]. While these treatments alleviate the motor symptoms of PD, they are not neuroprotective in nature and cannot stop disease progression or reverse the neurodegeneration. Moreover, long-term usage can cause serious side effects such as dyskinesia [9]. Therefore, many efforts have gone into understanding the pathogenesis of PD in order to develop neuroprotective treatments. Both environmental and genetic factors such as α-synuclein (SNCA) and leucine-rich repeat kinase 2 (LRRK2) mutations have been reported to contribute to PD development through regulating the mitochondrial function in DA neurons [10,11]. Blood-brain barrier (BBB) compromise and peripheral immune components infiltration have also been reported in PD patients [12,13]. These infiltrated immune components may interact with DA neurons and lead to DA neuronal demise directly. More recently, miRNAs have been revealed to be regulated by the PD risk genes and may contribute to PD through a direct regulation on the mitochondrial and immune pathways. 

## 2. Search Strategy/Methods

Using the advanced search builder function in PubMed, a systemic search was performed with keywords ‘“MicroRNAs” (Majr) and Parkinson’s disease’ in September 2019. This resulted in 253 titles consisting of both reviews and original research articles. All relevant articles including screening studies and mechanistic studies were examined to identify the promising microRNAs in PD that are discussed in this review.

## 3. The Biogenesis and Function of miRNAs

MicroRNAs are small non-coding RNAs that are transcribed from miRNA genes and intronic sequences as primary miRNAs (pri-miRNAs) and stem-loop precursor miRNAs (pre-miRNAs) respectively. In the nucleus, pri-miRNAs are further processed to form pre-miRNAs by the Drosha/DGCR8 complex. Pre-miRNAs are then exported out of the nucleus by Exportin-5. In the cytosol, pre-miRNAs are cleaved by Dicer to produce double-stranded mature miRNAs. The mature guide strand, about 20–22 nucleotides long, is then loaded onto Argonaute proteins to form the RNA-induced silencing complex (RISC). The mature miRNA is responsible for associating RISC to its target messenger RNAs (mRNAs) by binding at complementary sequences located usually at the 3′-UTR of the mRNA [14,15] and function as powerful regulators of gene expression. The specificity of the targeting is especially dependent on the seed sequence of the miRNA found at the 5′-end (bases 2–8) of the miRNA [16,17]. When miRNAs originate from the same predicted precursor, they are assigned names in the form of miR-1-5p (from the 5′-arm) and miR-1-3p (from the 3′-arm). If the relative abundance is known, they can also be named as miR-1 (predominantly-expressed product) and miR-1* (from the opposite arm of the precursor). Identical mature miRNAs that are expressed from different precursor sequences and genomic loci are assigned names in the form of miR-1-1 and miR-1-2. Closely related mature sequences are assigned lettered suffixes, for example, miR-1a and miR-1b would be expressed from precursors mir-1a and mir-1b, respectively [18]. The binding of miRNA to its targets results in translational repression and downregulation in target protein expression. 

**Figure 1:**
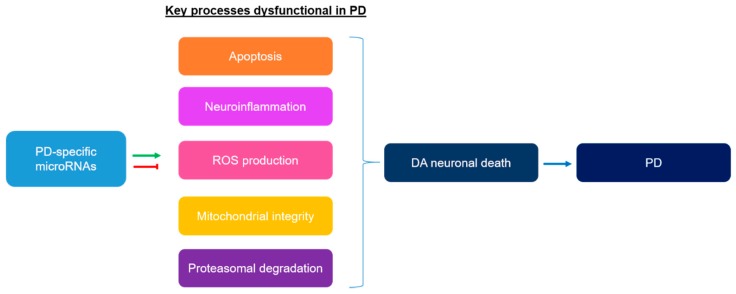
Key processes that are dysfunctional in Parkinson’s disease (PD) and could be regulated by a group of PD-specific differentially expressed miRNAs.

Since the discovery of miRNAs in 1993, increasing evidence have revealed miRNAs to be important biological molecules involved in diverse processes to maintain normal cellular functions [19,20,21]. In addition, extracellular miRNAs are relatively stable as they are protected from degradation by being bound to RNA-binding proteins and/or being packaged into exosomes [22], thus, revealing its potential as diagnostic biomarkers in diseases. The dysregulation of miRNAs may contribute to the development of various diseases from brain disorders to cancers [23,24]. In fact, many studies have shown that the miRNAs expression profile is dysregulated in PD and may contribute to PD pathogenesis. Figure 1 shows some of the key dysfunctional processes in PD that could be regulated by PD-specific miRNAs.

Using techniques such as RNA sequencing, microarray and microRNA qPCR profiling, many screening studies have been conducted in attempt to characterise the miRNAs dysregulation in PD in both the central nervous system (CNS) and the periphery. Our search strategy revealed 34 screening studies that have been conducted to identify the clinically significant miRNAs in PD. We included studies on animal PD models and in vitro neuronal models as we believe that clinically significant miRNAs identified in PD patients will also be highlighted in these models. Moreover, due to the difficulty in obtaining human PD patients’ samples, the inclusion of these model studies can also reveal suitable PD models to study mechanisms of the identified miRNA(s). In Table 1, we show the list of miRNAs that have been identified by more than 3 screening studies to be dysregulated in PD. Of the miRNAs listed in Table 1, 15 miRNAs have been reported to be dysregulated in all three areas; the brain/neuronal model(s), cerebrospinal fluid (CSF) and blood. In Table 2, we show the details of the studies on these 15 miRNAs, including the methods used, the PD models employed and the area(s) studied. 

The top 5 miRNAs listed in Table 2, namely, miR-30, miR-29, let-7, miR-485 and miR-26 will be reviewed and their possible roles in interaction with PD risk genes, mitochondrial function and immune pathways which may lead to PD progression will be discussed. The significance of miRNA research and the challenges faced in this field will be highlighted.

### 3.1. miR-30

The miR-30 family consists of 5 members and 6 mature miRNA sequences, namely, miR-30a, miR-30b, miR-30c-1, miR-30c-2, miR-30d and miR-30e. The members share a common seed sequence near the 5′-end but has different compensatory sequences near the 3′-end, allowing the different members to target different genes and pathways [57]. 

In the post-mortem human brain studies, miR-30b in the substantia nigra (SN) [25] and miR-30c-2 and miR-30d in cingulate gyri [26] were found to be upregulated in PD patients compared to healthy controls. Moreover, miR-30b was upregulated in exosomes isolated from the CSF of PD patients [30]. In addition, the dysregulation observed for miR-30c-2 was also found to be positively correlated in both the prefrontal cortex and blood leukocytes of PD patients [27], suggesting the potential of examining the miRNA profile of peripheral leukocytes of PD patients for diagnostic biomarkers.

Besides the CNS, differential expression levels of miR-30 family members have also been detected in the peripheral blood of PD patients as compared to healthy controls. Downregulated expressions of miR-30b and miR-30c in the PBMCs [31] and downregulated miR-30a in the plasma [32] were reported in PD patients. Contrary to these findings, miR-30a-5p and miR-30b-5p were reported to be upregulated in the plasma and WBCs of L-dopa-treated PD patients, respectively, while miR-30b-5p was upregulated in the plasma of drug naïve PD patients [33]. miR-30a-3p and miR-30e-3p were also found to be upregulated in the serum of PD patients [35]. Despite the discrepancies which can be improved through proper stratification of the patients by disease stage and drug treatment during patient recruitment, these discoveries suggest that miR-30 may play functional roles in PD progression.

Using a PD mouse model, Dorval and colleagues (2014) revealed that miR-30a* was upregulated in the striatal tissues of LRRK2-KO mice [28] which indicates that LRRK2 may function through regulating miRNAs expression. In addition, miR-30e has been reported to be neuroprotective in PD. Using the MPTP-induced PD mouse model, Li et al. (2018) found that the delivery of miR-30e agomir improves the mice’s motor behavioural performances on the rota-rod, pole and traction tests and the beam-crossing task. miR-30e agomir treatment was also able to attenuate TH loss induced by MPTP, decrease a-syn expression and restore brain-derived neurotrophic factor (BDNF) secretion. Li and colleagues also showed that miR-30e can ameliorate neuroinflammation by reducing inflammatory cytokines TNF-α, COX-2 and iNOS, and target Nlrp3 to inhibit the activation of Nlrp3 inflammasome [58]. Besides its role in regulating neuroinflammation, by comparing the expression profiles of PD patients with rapid and slow progression rates obtained from Gene Expression Omnibus GSE80599, Fan and Xiao (2018) identified a total of 225 differentially expressed genes that were significantly enriched in pathways related to fatty acid metabolism and may be regulated by the miR-30 family members [59]. On the other hand, miR-30a-5p has been reported to target and suppress BDNF expression [60]. Since BDNF contributes to the plasticity and repair of DA neurons [61,62] and a lower BDNF level is associated with greater cognitive impairments in PD patients [63], this suggests that miR-30a may play a neurotoxic role in PD.

### 3.2. miR-29

The miR-29 family consists of 3 members and 4 mature miRNA sequences, namely, miR-29a, miR-29b-1, miR-29b-2 and miR-29c. Mature sequences have identical seed sequences at nucleotide positions 2-7, suggesting that the targets for miR-29 members heavily overlap [64]. 

In the post-mortem human brain, miR-29a, miR-29b-1 and miR-29b-2 were observed to be upregulated in the anterior cingulate gyri of PD patients [26]. The dysregulation observed for miR-29b-2-5p was also found to be positively correlated in both the prefrontal cortex and blood leukocytes of PD patients [27]. In contrast, miR-29c was downregulated in the exosomes isolated from the CSF of PD patients [30].

In the periphery, miR-29a-3p was reported to be upregulated in the WBCs of L-dopa-treated PD patients but not in the untreated PD patients [33]. On the contrary, miR-29a [38] and miR-29c [37,38] levels were observed to be decreased in the serum of PD patients. In addition, miR-29b and miR-29c were downregulated in the PBMCs of PD patients [31] while decreased miR-29a was observed in the blood of both drug-treated and untreated PD patients as compared to healthy controls [39]. It was suggested that blood serum levels of miR-29a and miR-29c tended to decrease with PD severity [65].

miR-29 has been shown to regulate various processes that are important in PD development, such as apoptosis [66,67,68] and neuronal survival [67], cellular senescence [69,70], fine-tuning motor functions [67,71], immune regulation [72,73,74] and epigenetic modulation [75,76]. Kole et al. (2011) showed that miR-29b contributes to neuronal survival by targeting genes in the pro-apoptotic BH3-only family to inhibit apoptosis [66]. Moreover, Roshan et al. (2014) demonstrated that the inhibition of miR-29 in the mouse brain resulted in massive cell death especially in the hippocampus and cerebellum [67]. These mice also exhibited characteristics of ataxia such as reduced step length, which suggests a role for miR-29 in motor coordination [67]. In addition, miR-29 also modulates immune responses by regulating T helper 1 (Th1) differentiation and targeting transcription factors T-bet and Eomes, resulting in the repression of IFN-γ production [72]. Downregulation of miR-29 levels in the periphery of PD patients [31,37,38,39] supports the observation of naïve CD4+ T cells from the peripheral blood of PD patients to preferentially differentiate towards Th1 lineage [77]. These studies suggest that the dysregulation of miR-29 may mediate PD progression. 

### 3.3. let-7

The let-7 family members consist of let-7a to let-7-k, miR-98 and miR-202. While the let-7 sequence is well-conserved from nematode to human, different species can express different members of let-7. For example, in humans, we do not express let-7h/j/k. Interestingly, all let-7 members share the same seed sequence (nucleotides 2–8) for target recognition [78]. 

In post-mortem human brain studies, let-7b in the SN [25] and let-7e in the anterior cingulate gyri [26] were found to be upregulated in PD patients compared to healthy controls. Moreover, let-7g-3p was upregulated in the CSF of PD patients [30,35]. The dysregulation observed for let-7d-5p, let-7f-5p and let-7g were also found to be positively correlated in both the prefrontal cortex and blood leukocytes of PD patients [27]. In contrast, downregulated expressions of let-7a and let-7f were found in the plasma of treatment-naïve PD patients [32]. 

In animal PD models, let-7f was observed to be upregulated in the striatal tissues of LRRK2-KO mice as compared to control mice [28] while let-7 was reported to be downregulated in an A53T α-synuclein-overexpressed model of *Caenorhabditis elegans* (*C. elegans*) [40].

Extensive research conducted on let-7 revealed that let-7 members regulate processes such as apoptosis [79,80], immune system modulation [81,82,83,84,85,86], axon guidance [87] and regeneration [88,89], sleep [90], blood–brain-barrier maintenance [91] and cellular senescence [92]. Li and colleagues (2017) showed that the overexpression of let-7d promoted cell viability while let-7d-KO reduced cell viability in 6-OHDA-treated MN9D cells through the binding of let-7d to the 3′-UTR of caspase-3 mRNA, thus reducing the activity of caspase-3-mediated cell death [79]. In addition, let-7/miR-98 can target the 3′-UTR of Fas and reduce Fas-mediated apoptosis [80]. These evidence suggest that let-7 could be involved in regulating neuronal degeneration in PD. Besides apoptosis, let-7 has been shown to regulate several immune processes. In B cells, the let-7a-1/let-7d/let-7f-1 cluster (let-7adf) specifically inhibits T cell-independent (TI) antigen-induced immunoglobulin (Ig)M antibody production by reducing glucose and glutamine availability, two principal nutrients important for B cells activation. Jiang et al. (2018) showed that let-7adf can target Hexokinase 2 to suppress glycolysis as well as regulate c-Myc to suppress glutamine uptake, thus demonstrating the adaptive immune role of let-7adf as a metabolic brake on B cell antibody production [81]. Another study showed that the overexpression of let-7c can promote macrophage polarization from M1 to M2 phenotype and regulate bactericidal and phagocytic activities of macrophages [82]. let-7 also plays a role in facilitating CD4 T cells anergy [83] as well as the proliferation and differentiation of CD8 T cells [84]. In AD, it has been shown that let-7 is overexpressed and released [85]. Moreover, the extracellular let-7 can act as endogenous damage-associated molecular patterns (DAMPs) and be recognised by TLR7 to promote inflammation and neuronal death [85]. Furthermore, let-7 is able to modulate inflammation by repressing production of cytokines IL-6 and IL-10 [86]. Let-7 also has PD-specific functions. LRRK2 is able to inhibit let-7 and miR-184* expression, leading to the overexpression of target genes E2F1 and DP, respectively. Since these genes contribute to cell cycle and survival regulation, E2F1/DP dysregulation is important for mediating toxic effects similar to pathogenic LRRK2 such as the degeneration of dopaminergic neurons [93]. In addition, the loss of let-7 can lead to decreased α-synuclein expression and accumulation, increased autophagy, increased oxidative stress and increased lipid content in *C. elegans* [94]. Therefore, the targeting of let-7 or its targets may have therapeutic potential in PD treatment. 

### 3.4. miR-485

Little has been discovered about the functions of miR-485. However, this miRNA has been suggested to be dysregulated in PD. In the post-mortem human brain, miR-485-5p was reported to be downregulated in the SN [43] while the complementary strand, miR-485-3p was observed to be upregulated in the putamen tissues [44] of PD patients as compared to healthy controls. Conflicting trends were also observed in the CSF. Gui et al. (2015) reported upregulation of miR-485-5p in the exosomes isolated from the CSF of PD patients [30] while Burgos et al. (2014) showed that miR-485-5p was downregulated in the CSF of PD patients [35]. In the plasma, miR-485-5p was found to be downregulated in PD patients [32,45]. 

Some miR-485-mediated pathways suggested in the literature include apoptosis [95,96], immune modulation [97,98], iron homeostasis [99] and synaptic plasticity [100]. miR-485-5p can target matrix metalloprotease (MMP)-14 [97], a membrane protein that can promote axon regeneration when inhibited [101]. MMP-14 upregulation is also observed in microglia/macrophages associated with neurodegenerative and neuroinflammatory pathologies both in human biopsies and mouse models [98]. In AD, the expression of MMP-14 was found in reactive astrocytes [102]. These evidences suggest that miR-485 can regulate immune responses through MMP-14. In contrast, conflicting observations were reported for miR-485 in regulating apoptosis. Wang et al. (2018) demonstrated that miR-485 can target Smad ubiquitin regulatory factor 2 (Smurf2) and promote apoptosis through the Smurf2-mediated TGF-β/Smads signalling pathway in chronic asthmatic mouse models [95]. However, Chen and colleagues (2016) showed that miR-485-5p is able to target TNF receptor (TNFR)-associated death domain (TRADD) and prevent TNF-α-induced neuronal cell apoptosis [96]. 

Beside apoptosis, miR-485 is also able to target the expression of peroxisome proliferator-activated receptor gamma (PPARγ) coactivator-1α (PGC-1α) [103]. PGC-1α can induce the expression of reactive oxygen species (ROS) scavenging enzymes, such as, glutathione peroxidase-1, catalase and superoxide dismutase, to reduce oxidative stress [104]. Moreover, PGC-1α knockout mice displayed an increased vulnerability to MPTP-induced degeneration of nigral DA neurons [104]. PGC-1α knockdown also increased α-synuclein accumulation in neuronal cells [105], suggesting the neuroprotective role for PGC-1α. Thus, the suppression of PGC-1α by miR-485 indicates a neurotoxic function for miR-485 in PD.

### 3.5. miR-26

The miR-26 family consists of miR-26a-1, miR-26a-2 and miR-26b in humans [106]. In the CNS, miR-26a was observed to be upregulated the SN tissues and in the exosomes isolated from the CSF of PD patients as compared to healthy controls [25,30]. In the striatal tissues of rodent PD models, miR-26a upregulation was observed in rotenone-treated rats [47] while miR-26b upregulation was reported in LRRK2-KO mice [28]. However, in the PBMCs isolated from PD patients, miR-26a downregulation was detected instead [31]. 

The role of miR-26 in neurodegenerative diseases has not been well-studied. However, in other disease models, miR-26 has been suggested to modulate processes such as DNA repair [107,108], apoptosis [109,110], autophagy [111,112], LTP maintenance [113], immune regulation [114,115] and glucose and lipids metabolism [116]. miR-26a is able to target phosphatase and tensin homolog on chromosome 10 (PTEN) [107]. PTEN has both protein and lipid phosphatase activity that inhibits the PI3K/AKT pathway involved in cell survival [108]. Thus, overexpression of PTEN may contribute to the activation of proteolytic cascade for apoptosis [117]. In addition, PTEN-induced kinase 1 (PINK1) has been demonstrated to be important in safeguarding mitochondrial function and integrity and mutations in PINK1 have been linked to familial PD [118]. Thus, miR-26 may regulate neurodegeneration in PD through PTEN and PINK1. Besides PTEN, miR-26 can also induce apoptosis and inhibit autophagy by targeting TGF-β and suppressing the TGF-β/JNK signalling pathway [111]. miR-26 may also modulate the immune system through NF-κB signalling. miR-26 is able to dampen the induction of cytokines, such as IL-6, by downregulating the TNF-α/NF-κB signalling axis through the silencing of 2 NF-κB signalling factors, HMGA1 and MALT1 [114]. Furthermore, miR-26b can directly target TAK1 and TAB3, the upstream positive regulators of the NF-κB pathway [115]. Thus, these evidence suggest that miR-26 may play a role in suppressing neuroinflammation in PD.

## 4. Significance and Challenges of miRNA Research

We have highlighted several candidate miRNAs that may aggravate or mitigate PD progression through their actions in the CNS or periphery. miRNAs are capable of regulating several signaling pathways as each miRNA can target and bind to an average of 100–200 genes, making them potent modulators of gene expression [119]. Therefore, it is important to uncover the mechanisms of dysregulated miRNAs in diseases as these miRNAs and their targets can have great therapeutic potential in the development of treatments for these diseases. In addition, the presence of dysregulated miRNAs in blood also signal the prospect of using serum/plasma miRNAs as diagnostic markers for diseases. The success of miRNA biomarker research could translate to more accurate disease diagnosis using less invasive methods. This is especially important in neurodegenerative diseases, such as PD, where diagnosis is often given based on symptomatic examinations and cognitive assessments rather than through more objective tests (such as blood tests). 

Despite the importance of miRNA research, the literature currently available on the actions of miRNA in PD is greatly lacking. Moreover, results of miRNA studies have not been consistent with each other. There are several potential limitations in published studies. It is difficult to identify clinically important miRNAs in diseases because clinical studies thus far are limited by their small sample size which may not have sufficient power to identify the effect size difference. In addition, the areas investigated also differ in the studies as noted in Table 2. Moreover, the techniques used to characterise the miRNA profile in each study have different sensitivities which can affect the output data obtained. The lack of a standardised protocol in miRNA isolation and detection has led to the identification of non-overlapping and non-replicating sets of potentially dysregulated miRNAs. Factors such as different study designs and experimental conditions, different tissue sources or PD models and the use of PD samples with different sample size, clinical features and pharmacological treatment could all result in discordant results among studies. Therefore, clinical articles published on the same disease may report very different miRNA expression profiles, making it difficult to identify or validate biologically relevant miRNAs.

There are also several challenges in developing miRNA-based therapeutic therapies. One major problem is the delivery of candidate miRNA(s) to specific sites. The development of specific and effective miRNA delivery systems is vital as the delivery vehicle must allow miRNAs to cross the blood–brain barrier. As miRNAs are easily degradable, the delivery systems employed must also be able to stabilise and extend the life of the miRNAs. Furthermore, using miRNAs in treatment may be a double-edged sword. While it is advantageous that miRNAs as powerful modulators of gene expression have the ability to alter several signaling pathways at once to switch the cellular physiology from an apoptotic state to one that favors survival, this also means that side effects in unspecific sites could be problematic. Hence, specific and effective delivery systems in miRNA-based therapy are vital. The current experimental approaches used in miRNA research have many limitations. As miRNAs are small and easily degradable, the existing isolation methods can lead to a great loss in yield or a biased recovery of certain miRNAs. This can affect the potential of miRNAs as diagnostic markers for diseases. Moreover, the experimental approaches required for miRNA studies are also very costly. Hence, these challenges could slow down the progression of miRNAs research. 

## 5. Conclusions

We have highlighted several miRNAs that show promise in having therapeutic and/or diagnostic potential in PD as well as the pathophysiologic role of miRNAs in disease states. Developing an optimal delivery system and unravelling druggable targets of miRNAs in both experimental and human models and clinical validation of the results may pave the way for novel therapeutics in PD.

## Figures and Tables

**Table 1:** Summary of dysregulated miRNAs in PD identified by more than 3 screening studies.

miRNA	No. of Studies	Brain Region/Neuronal Model	CSF	Blood
miR-30	11	Briggs et al., 2015 [25]; Tatura et al., 2016 [26]; Chatterjee and Roy, 2017 [27]; Dorval et al., 2014 [28]; He et al., 2017 [29]	Gui et al., 2015 [30]	Chatterjee and Roy, 2017 [27]; Martins et al., 2011 [31]; Chen et al., 2018 [32]; Schwienbacher et al., 2017 [33]; Serafin et al., 2015 [34]; Burgos et al., 2014 [35]
miR-29	10	Tatura et al., 2016 [26]; Lungu et al., 2013 [36]; Chatterjee and Roy, 2017 [27]	Gui et al., 2015 [30]	Chatterjee and Roy, 2017 [27]; Serafin et al., 2015 [34]; Schwienbacher et al., 2017 [33]; Ma et al., 2016 [37]; Martins et al., 2011 [31]; Botta-Orfila et al., 2014 [38]; Margis et al., 2011 [39]
let-7	9	Briggs et al., 2015 [25]; Tatura et al., 2016 [26]; Chatterjee and Roy, 2017 [27]; Dorval et al., 2014 [28]; Asikainen et al., 2010 [40]; He et al., 2017 [29]	Burgos et al., 2014 [35]; Gui et al., 2015 [30]	Chatterjee and Roy, 2017 [27]; Chen et al., 2018 [32]
miR-19	6	-	Burgos et al., 2014 [35]; Gui et al., 2015 [30]	Martins et al., 2011 [31]; Cao et al., 2017 [41]; Botta-Orfila et al., 2014 [38]; Rosas-Hernandez et al., 2018 [42]
miR-485	6	Cardo et al., 2014 [43]; Nair and Ge, 2016 [44]	Burgos et al., 2014 [35]; Gui et al., 2015 [30]	Khoo et al., 2012 [45]; Chen et al., 2018 [32]
miR-132	5	Briggs et al., 2015 [25]; Lungu et al., 2013 [36]; Nelson et al., 2018 [46]	Burgos et al., 2014 [35]; Gui et al., 2015 [30]	-
miR-26	5	Briggs et al., 2015 [25]; Horst et al., 2018 [47]; Dorval et al., 2014 [28]	Gui et al., 2015 [30]	Martins et al., 2011 [31]
miR-221	5	Tatura et al., 2016 [26]; Nair and Ge, 2016 [44]; Chatterjee and Roy, 2017 [27]	-	Chatterjee and Roy, 2017 [27]; Ma et al., 2016 [37]; Ding et al., 2016 [48]
miR-181	5	Briggs et al., 2015 [25]; Mo et al., 2017 [49]; Chatterjee and Roy, 2017 [27]; Lungu et al., 2013 [36]	-	Chatterjee and Roy, 2017 [27]; Ding et al., 2016 [48]
miR-200	4	He et al., 2017 [29]; Mo et al., 2017 [49]	Mo et al., 2017 [49]	Khoo et al., 2012 [45]; Chen et al., 2018 [32]
miR-133	4	Briggs et al., 2015 [25]; Kong et al., 2015 [50]; Nelson et al., 2018 [46]	-	Rosas-Hernandez et al., 2018 [42]
miR-16	4	Briggs et al., 2015 [25]; Dorval et al., 2014 [28]	Gui et al., 2015 [30]	Burgos et al., 2014 [35]
miR-185	4	Lungu et al., 2013 [36]; Nelson et al., 2018 [46]	-	Chen et al., 2018 [32]; Ding et al., 2016 [48]
miR-126	4	Briggs et al., 2015 [25]	Gui et al., 2015 [30]	Martins et al., 2011 [31]; Rosas-Hernandez et al., 2018 [42]
miR-146	4	Lungu et al., 2013 [36]; Dorval et al., 2014 [28]	-	Ma et al., 2016 [37]; Dong et al., 2016 [51]
miR-218	4	Briggs et al., 2015 [25]; He et al., 2017 [29]; Nelson et al., 2018 [46]; Chatterjee and Roy, 2017 [27]	-	Chatterjee and Roy, 2017 [27]
miR-548	4	Cardo et al., 2014 [43]; Tatura et al., 2016 [26]; Chatterjee and Roy, 2017 [27]	-	Chatterjee and Roy, 2017 [27]; Khoo et al., 2012 [45]
miR-671	4	He et al., 2017 [29]; Dorval et al., 2014 [28]; Chatterjee and Roy, 2017 [27]	-	Chatterjee and Roy, 2017 [27]; Khoo et al., 2012 [45]
miR-330	4	Briggs et al., 2015 [25]; Lungu et al., 2013 [36]; Cardo et al., 2014 [43]; Chatterjee and Roy, 2017 [27]	-	Chatterjee and Roy, 2017 [27]
miR-135	4	Briggs et al., 2015 [25]; Nelson et al., 2018 [46]; Cardo et al., 2014 [43]; Lungu et al., 2013 [36]	-	-
miR-331	4	Briggs et al., 2015 [25]; Chatterjee and Roy, 2017 [27]	Gui et al., 2015 [30]	Chatterjee and Roy, 2017 [27]; Cardo et al., 2013 [52]
miR-10	4	Tatura et al., 2016 [26]; Mo et al., 2017 [49]	Gui et al., 2015 [30]; Burgos et al., 2014 [35]	-
miR-24	4	Briggs et al., 2015 [25]; Chatterjee and Roy, 2017 [27]	Marques et al., 2017 [53]	Chatterjee and Roy, 2017 [27]; Cao et al., 2017 [41]
miR-151	4	He et al., 2017 [29]; Chatterjee and Roy, 2017 [27]	Gui et al., 2015 [30]	Chatterjee and Roy, 2017 [27]; Martins et al., 2011 [31]
miR-125	4	Briggs et al., 2015 [25]; Dorval et al., 2014 [28]; Chatterjee and Roy, 2017 [27]	-	Chatterjee and Roy, 2017 [27]; Chen et al., 2018 [32]
miR-99	4	Tatura et al., 2016 [26]; Chatterjee and Roy, 2017 [27]	-	Chatterjee and Roy, 2017 [27]; Khoo et al., 2012 [45]; Sheinerman et al., 2017 [54]
miR-106	3	Briggs et al., 2015 [25]; Lungu et al., 2013 [36]; Chatterjee and Roy, 2017 [27]	-	Chatterjee and Roy, 2017 [27]
miR-34	3	Briggs et al., 2015 [25]; Horst et al., 2018 [47]; Nelson et al., 2018 [46]	-	-
miR-124	3	Briggs et al., 2015 [25]; Tatura et al., 2016 [26]	-	Rosas-Hernandez et al., 2018 [42]
miR-193	3	Briggs et al., 2015 [25]; Mo et al., 2017 [49]	-	Dong et al., 2016 [51]
miR-27	3	Briggs et al., 2015 [25]; Dorval et al., 2014 [28]	-	Chen et al., 2018 [32]
miR-338	3	Briggs et al., 2015 [25]; Nelson et al., 2018 [46]	-	Burgos et al., 2014 [35]
miR-214	3	Dorval et al., 2014 [28]	-	Ma et al., 2016 [37]; Dong et al., 2016 [51]
miR-7	3	Tatura et al., 2016 [26]; Nelson et al., 2018 [46]; Horst et al., 2018 [47]	-	-
miR-219	3	Briggs et al., 2015 [25]; He et al., 2017 [29]; Nair and Ge, 2016 [44]	-	-
miR-299	3	Tatura et al., 2016 [26]; Dorval et al., 2014 [28]; Cardo et al., 2014 [43]	-	-
miR-320	3	Briggs et al., 2015 [25]; He et al., 2017 [29]; Chatterjee and Roy, 2017 [27]	-	Chatterjee and Roy, 2017 [27]
miR-543	3	Tatura et al., 2016 [27]; Nelson et al., 2018 [46]; Mo et al., 2017 [49]	-	-
miR-136	3	Chatterjee and Roy, 2017 [27]	Gui et al., 2015 [30]; Burgos et al., 2014 [35]	Chatterjee and Roy, 2017 [27]
miR-1	3	Asikainen et al., 2010 [40]	Gui et al., 2015 [30]	Margis et al., 2011 [39]
miR-374	3	Briggs et al., 2015 [25]	Gui et al., 2015 [30]	Martins et al., 2011 [31]
miR-28	3	Briggs et al., 2015 [25]	Gui et al., 2015 [30]	Martins et al., 2011 [31]
miR-301	3	He et al., 2017 [29]	Gui et al., 2015 [30]	Martins et al., 2011 [31]
miR-129	3	Tatura et al., 2016 [26]; He et al., 2017 [29]	-	Sheinerman et al., 2017 [54]
miR-9	3	Briggs et al., 2015 [25]	-	Khoo et al., 2012 [45]; Sheinerman et al., 2017 [54]
miR-92	3	Briggs et al., 2015 [25]; He et al., 2017 [29]; Chatterjee and Roy, 2017 [27]	-	Chatterjee and Roy, 2017 [27]
miR-15	3	Dorval et al., 2014 [28]; Lungu et al., 2013 [36]	-	Ding et al., 2016 [48]
miR-378	3	Mo et al., 2017 [49]; He et al., 2017 [29]; Chatterjee and Roy, 2017 [27]	-	Chatterjee and Roy, 2017 [27]
miR-222	3	He et al., 2017 [29]	-	Khoo et al., 2012 [45]; Chen et al., 2018 [32]
miR-874	3	Dorval et al., 2014 [28]	-	Chen et al., 2018 [32]; Sheinerman et al., 2017 [54]

**Table 2:** Summary of 15 miRNAs reported to be dysregulated in the brain/neuronal model(s), cerebrospinal fluid (CSF) and blood.

miRNA Family	miRNA Member	Trend	Brain Region/Neuronal Model	CSF	Blood	Method	Ref.
miR-30	hsa-miR-30b	↑	SN tissues from 8 controls vs 8 PD patients (5 males and 3 females in each group)	-	-	Human MicroRNA TaqMan Arrays A 2.0	[25]
	hsa-miR-30c-2, -30d	↑	Anterior cingulate gyri from 10 controls vs. 22 PD patients	-	-	TaqMan Array MicroRNA cards (pools A and B)	[26]
	hsa-miR-30c-2	- ^#^	Prefrontal cortex from 33 controls vs. 29 PD patients	-	Leukocytes from 6 controls vs. 7 PD patients (All males)	Comparative analysis of GSE72962 (non-coding RNA sequencing) and GSE40915 (non-coding RNA sequencing)	[27,55,56]
	mmu-miR-30a*	↑	Striatal tissues of LRRK2-knockout mice vs. controls (4 per group)	-	-	Mouse Gene 1.0 ST and miRNA (v1 or v2) microarrays (Affymetrix)	[28]
	hsa-miR-30c-1-3p	↓	MnCl_2_-treated SH-SY5Y cells	-	-	Small RNA sequencing (Illumina HiSeq2000)	[29]
	hsa-miR-30b	↑	-	Exosomes isolated from CSF from 27 controls vs. 47 PD patients	-	TaqMan Low-Density Array Human miRNA Panels (pool A and B)	[30]
	hsa-miR-30b, -30c	↓	-	-	PBMCs of 13 controls and 19 PD patients	Exiqon-developed miRCURY LNA array (version 10.0)	[31]
	hsa-miR-30a	↓	-	-	Plasma from 25 controls vs. 25 drug-naïve PD patients	A customized neurodegenerative disease-related 91 miRNA panel prepared by miRGenes	[32]
	hsa-miR-30a-5p	↑	-	-	Plasma from 99 L-dopa-treated PD patients vs. control pairs	qPCR	[33]
	hsa-miR-30b-5p	↑	-	-	Plasma from 10 drug-naive PD patients vs. control pairs	qPCR	[33]
	hsa-miR-30b-5p	↑	-	-	WBCs from 36 L-dopa-treated PD patients vs. control pairs	qPCR	[33,34]
	hsa-miR-30a-3p, -30e-3p	↑	-	-	Serum from 60 PD patients vs. 72 controls	Illumina TruSeq Small RNA sequencing (Illumina HiSeq2000)	[35]
miR-29	hsa-miR-29a, -29b-1, -29b-2	↑	Anterior cingulate gyri from 10 controls vs. 22 PD patients	-	-	TaqMan Array MicroRNA cards (pools A and B)	[26]
	rno-miR-29a-3p, -29b-2-5p	↑	Mesencephalon brain areas from BD-IV affected rats (n=3) vs. control BD-IV rats (n=3)	-	-	Microarray (Chip ID miRRat 19.0 version)	[36]
	hsa-miR-29b-2-5p	- ^#^	Prefrontal cortex from 33 controls vs. 29 PD patients	-	Leukocytes from 6 controls vs. 7 PD patients (All males)	Comparative analysis of GSE72962 (non-coding RNA sequencing) and GSE40915 (non-coding RNA sequencing)	[27,55,56]
	hsa-miR-29c	↓	-	Exosomes isolated from CSF from 27 controls vs. 47 PD patients	-	TaqMan Low-Density Array Human miRNA Panels (pool A and B)	[30]
	hsa-miR-29a-3p	↑	-	-	WBCs from 36 L-dopa-treated PD patients vs. control pairs	qPCR	[33,34]
	hsa-miR-29c	↓	-	-	Serum from 138 PD patients vs. 112 controls	qPCR	[37]
	hsa-miR-29b, -29c	↓	-	-	PBMCs of 13 controls and 19 PD patients	Exiqon-developed miRCURY LNA array (version 10.0)	[31]
	hsa-miR-29a, -29c	↓	-	-	Serum from 65 idopathic PD patients vs. 65 controls	TaqMan Array MicroRNA A Cards v2.0 followed by qPCR	[38]
	hsa-miR-29a	↓	-	-	Blood from 8 untreated PD patients, 4 drug-treated PD patients vs. 8 controls	qPCR	[39]
let-7	hsa-let-7b	↑	SN tissues from 8 controls vs. 8 PD patients (5 males and 3 females in each group)	-	-	Human MicroRNA TaqMan Arrays A 2.0	[25]
	hsa-let-7e	↑	Anterior cingulate gyri from 10 controls vs. 22 PD patients	-	-	TaqMan Array MicroRNA cards (pools A and B)	[26]
	hsa-let-7d-5p, -7f-5p, -7g	- ^#^	Prefrontal cortex from 33 controls vs. 29 PD patients	-	Leukocytes from 6 controls vs. 7 PD patients (All males)	Comparative analysis of GSE72962 (non-coding RNA sequencing) and GSE40915 (non-coding RNA sequencing)	[27,55,56]
	mmu-let-7f	↑	Striatal tissues of LRRK2-knockout mice vs. controls (4 per group)	-	-	Mouse Gene 1.0 ST and miRNA (v1 or v2) microarrays (Affymetrix)	[28]
	cel-let-7	↓	A53T α-synuclein transgenic *C. elegans* vs. wildtype	-	-	Ncode Multispecies miRNA Microarray V2-arrays	[40]
	hsa-let-7f-1-3p	↓	MnCl_2_-treated SH-SY5Y cells	-	-	Small RNA sequencing (Illumina HiSeq2000)	[29]
	hsa-let-7g-3p	↑	-	CSF from 65 PD patients vs. 70 controls	-	Illumina TruSeq Small RNA sequencing (Illumina HiSeq2000)	[35]
	hsa-let-7g-3p	↑	-	Exosomes isolated from CSF from 27 controls vs. 47 PD patients	-	TaqMan Low-Density Array Human miRNA Panels (pool A and B)	[30]
	hsa-let-7a, -7f	↓	-	-	Plasma from 25 controls vs. 25 drug-naïve PD patients	A customized neurodegenerative disease-related 91 miRNA panel prepared by miRGenes	[32]
miR-485	hsa-miR-485-5p	↓	SN tissues from 4 controls vs. 8 PD patients	-	-	TaqMan low-density array	[43]
	hsa-miR-485-3p	↑	Putamen tissues from 12 PD patients vs. 12 controls	-	-	nCounter Human v2 miRNA Expression Assay kit	[44]
	hsa-miR-485-5p	↑	-	Exosomes isolated from CSF from 27 controls vs. 47 PD patients	-	TaqMan Low-Density Array Human miRNA Panels (pool A and B)	[30]
	hsa-miR-485-5p	↓	-	CSF from 65 PD patients vs. 70 controls	-	Illumina TruSeq Small RNA sequencing (Illumina HiSeq2000)	[35]
	hsa-miR-485-5p	↓	-	-	Plasma from 32 PD patients vs. 32 controls	Agilent whole human genome miRNA microarray v.3	[45]
	hsa-miR-485-5p	↓	-	-	Plasma from 25 controls vs. 25 drug-naïve PD patients	A customized neurodegenerative disease-related 91 miRNA panel prepared by miRGenes	[32]
miR-26	hsa-miR-26a-5p	↑	SN tissues from 8 controls vs. 8 PD patients (5 males and 3 females in each group)	-	-	Human MicroRNA TaqMan Arrays A 2.0	[25]
	rno-miR-26a	↑	Striatal tissues from rotenone-induced Wistar rats vs. control	-	-	qPCR	[47]
	mmu-miR-26b	↑	Striatal tissues of LRRK2-knockout mice vs. controls (4 per group)	-	-	Mouse Gene 1.0 ST and miRNA (v1 or v2) microarrays (Affymetrix)	[28]
	hsa-miR-26a	↑	-	Exosomes isolated from CSF from 27 controls vs. 47 PD patients	-	TaqMan Low-Density Array Human miRNA Panels (pool A and B)	[30]
	hsa-miR-26a	↓	-	-	PBMCs of 13 controls and 19 PD patients	Exiqon-developed miRCURY LNA array (version 10.0)	[31]
miR-200	hsa-miR-200a-5p	↑	MnCl_2_-treated SH-SY5Y cells	-	-	Small RNA sequencing (Illumina HiSeq2000)	[29]
	mmu-miR-200b-3p, -200a-3p, -200c-3p, 200a-5p	↑	Midbrain tissues of A53T α -synuclein-transgenic mice vs. wildtype	-	-	Illumina TruSeq Small RNA sequencing (Illumina HiSeq2500)	[49]
	hsa-miR-200a-3p	↑	-	CSF from 44 PD patients vs. 42 controls	-	qPCR	[49]
	hsa-miR-200a	↑	-	-	Plasma from 32 PD patients vs. 32 controls	Agilent whole human genome miRNA microarray v.3	[45]
	hsa-miR-200a	↓	-	-	Plasma from 25 controls vs. 25 drug-naïve PD patients	A customized neurodegenerative disease-related 91 miRNA panel prepared by miRGenes	[32]
miR-16	hsa-miR-16	↑	SN tissues from 8 controls vs. 8 PD patients (5 males and 3 females in each group)	-	-	Human MicroRNA TaqMan Arrays A 2.0	[25]
	mmu-miR-16	↑	Striatal tissues of LRRK2-knockout mice vs. controls (4 per group)	-	-	Mouse Gene 1.0 ST and miRNA (v1 or v2) microarrays (Affymetrix) followed by qPCR validation	[28]
	hsa-miR-16-2	↑	-	Exosomes isolated from CSF from 27 controls vs. 47 PD patients	-	TaqMan Low-Density Array Human miRNA Panels (pool A and B)	[30]
	hsa-miR-16-2-3p	↓	-	-	Serum from 60 PD patients vs. 72 controls	Illumina TruSeq Small RNA sequencing (Illumina HiSeq2000)	[35]
miR-126	hsa-miR-126	↑	SN tissues from 8 controls vs. 8 PD patients (5 males and 3 females in each group)	-	-	Human MicroRNA TaqMan Arrays A 2.0	[25]
	hsa-miR-126	↓	-	Exosomes isolated from CSF from 27 controls vs. 47 PD patients	-	TaqMan Low-Density Array Human miRNA Panels (pool A and B)	[30]
	hsa-miR-126*, -126	↓	-	-	PBMCs of 13 controls and 19 PD patients	Exiqon-developed miRCURY LNA array (version 10.0)	[31]
	mmu-miR-126a	↓	-	-	Serum from MPTP-treated mice vs. control	Next generation sequencing	[42]
miR-331	hsa-miR-331-3p	↑	SN tissues from 8 controls vs. 8 PD patients (5 males and 3 females in each group)	-	-	Human MicroRNA TaqMan Arrays A 2.0	[25]
	hsa-miR-331	- ^#^	Prefrontal cortex from 33 controls vs. 29 PD patients	-	Leukocytes from 6 controls vs. 7 PD patients (All males)	Comparative analysis of GSE72962 (non-coding RNA sequencing) and GSE40915 (non-coding RNA sequencing)	[27,55,56]
	hsa-miR-331-5p	↑	-	Exosomes isolated from CSF from 27 controls vs. 47 PD patients	-	TaqMan Low-Density Array Human miRNA Panels (pool A and B)	[30]
	hsa-miR-331-5p	↑	-	-	Plasma from 25 controls vs. 31 PD patients	TaqMan low density miRNA cards followed by qPCR validation	[52]
miR-24	hsa-miR-24	↑	SN tissues from 8 controls vs. 8 PD patients (5 males and 3 females in each group)	-	-	Human MicroRNA TaqMan Arrays A 2.0	[25]
	hsa-miR-24-3p	- ^#^	Prefrontal cortex from 33 controls vs. 29 PD patients	-	Leukocytes from 6 controls vs. 7 PD patients (All males)	Comparative analysis of GSE72962 (non-coding RNA sequencing) and GSE40915 (non-coding RNA sequencing)	[27,55,56]
	hsa-miR-24	↓	-	CSF from 28 PD patients vs. 28 controls	-	qPCR	[53]
	hsa-miR-24	↑	-	-	Exosomes isolated from serum of 109 PD patients vs. 40 controls	qPCR	[41]
miR-151	hsa-miR-151b	↓	MnCl_2_-treated SH-SY5Y cells	-	-	Small RNA sequencing (Illumina HiSeq2000)	[29]
	hsa-miR-151a-5p, -151b	- ^#^	Prefrontal cortex from 33 controls vs. 29 PD patients	-	Leukocytes from 6 controls vs. 7 PD patients (All males)	Comparative analysis of GSE72962 (non-coding RNA sequencing) and GSE40915 (non-coding RNA sequencing)	[27,55,56]
	hsa-miR-151	↓	-	Exosomes isolated from CSF from 27 controls vs. 47 PD patients	-	TaqMan Low-Density Array Human miRNA Panels (pool A and B)	[30]
	hsa-miR-151-5p, -151-3p	↓	-	-	PBMCs of 13 controls and 19 PD patients	Exiqon-developed miRCURY LNA array (version 10.0)	[31]
miR-1	cel-miR-1	↓	A53T α-synuclein transgenic *C. elegans* vs. wildtype	-	-	Ncode Multispecies miRNA Microarray V2-arrays	[40]
	hsa-miR-1	↓	-	Exosomes isolated from CSF from 27 controls vs. 47 PD patients	-	TaqMan Low-Density Array Human miRNA Panels (pool A and B)	[30]
	hsa-miR-1	↓	-	-	Blood from 8 untreated PD patients vs. 8 controls	qPCR	[39]
miR-374	hsa-miR-374a	↑	SN tissues from 8 controls vs. 8 PD patients (5 males and 3 females in each group)	-	-	Human MicroRNA TaqMan Arrays A 2.0	[25]
	hsa-miR-374	↓	-	Exosomes isolated from CSF from 27 controls vs. 47 PD patients	-	TaqMan Low-Density Array Human miRNA Panels (pool A and B)	[30]
	hsa-miR-374a, -374b	↓	-	-	PBMCs of 13 controls and 19 PD patients	Exiqon-developed miRCURY LNA array (version 10.0)	[31]
miR-28	hsa-miR-28-5p	↑	SN tissues from 8 controls vs. 8 PD patients (5 males and 3 females in each group)	-	-	Human MicroRNA TaqMan Arrays A 2.0	[25]
	hsa-miR-28	↓	-	Exosomes isolated from CSF from 27 controls vs. 47 PD patients	-	TaqMan Low-Density Array Human miRNA Panels (pool A and B)	[30]
	hsa-miR-28-5p	↓	-	-	PBMCs of 13 controls and 19 PD patients	Exiqon-developed miRCURY LNA array (version 10.0)	[31]
miR-301	hsa-miR-301a-5p	↓	MnCl_2_-treated SH-SY5Y cells	-	-	Small RNA sequencing (Illumina HiSeq2000)	[29]
	hsa-miR-301a	↓	-	Exosomes isolated from CSF from 27 controls vs. 47 PD patients	-	TaqMan Low-Density Array Human miRNA Panels (pool A and B)	[30]
	hsa-miR-301a	↓	-	-	PBMCs of 13 controls and 19 PD patients	Exiqon-developed miRCURY LNA array (version 10.0)	[31]

^#^ A positive correlation of the identified dysregulated miRNA was found in both the prefrontal cortex and the blood leukocytes of PD patients. ↑An upregulation in trend observed. ↓A downregulation in trend observed.
